# Predicting Water Flux in Forward Osmosis with Unknown Feed Solution Composition: An Empirical Approach Based on Thermodynamical Properties

**DOI:** 10.3390/membranes13040427

**Published:** 2023-04-12

**Authors:** Bastian Greisner, Dieter Mauer, Frank Rögener, André Lerch

**Affiliations:** 1 MionTec GmbH, 51377 Leverkusen, Germany; 2 TH Köln, Institute of Chemical Process Engineering and Plant Design, 50679 Köln, Germany; 3 Institute of Urban and Industrial Water Management, Technische Universität Dresden, 01062 Dresden, Germany

**Keywords:** membrane process, forward osmosis, simulation, water flux, unknown solution

## Abstract

This study investigated the predictability of forward osmosis (FO) performance with an unknown feed solution composition, which is important in industrial applications where process solutions are concentrated but their composition is unknown. A fit function of the unknown solution’s osmotic pressure was created, correlating it with the recovery rate, limited by solubility. The osmotic concentration was derived and used in the subsequent simulation of the permeate flux in the considered FO membrane. For comparison, magnesium chloride and magnesium sulfate solutions were used since these show a particularly strong deviation from the ideal osmotic pressure according to Van’t Hoff and are, thus, characterized by an osmotic coefficient unequal to 1. The simulation is based on the solution–diffusion model with consideration of external and internal concentration polarization phenomena. Here, a membrane module was subdivided into 25 segments of equal membrane area, and the module performance was solved by a numerical differential. Experiments in a laboratory scale for validation confirmed that the simulation gave satisfactory results. The recovery rate in the experimental run could be described for both solutions with a relative error of less than 5%, while the calculated water flux as a mathematical derivative of the recovery rate showed a bigger deviation.

## 1. Introduction

Due to rising energy prices, the demand for innovative energy-saving and sustainable processes is growing [1]. One focus of research and development is set on pressure-driven concentrating processes to be used instead of energy-intensive thermal processes such as evaporation [2,3]. In addition to reverse osmosis, which is a state-of-the-art approach in applications such as seawater desalination, more and more feasibility studies are being conducted on the use of forward osmosis (FO) [4]. In this context, FO is rather a useful supplement than a substitute for reverse osmosis. FO would, for example, be utilized where the feed has a high organic content with a high fouling potential [5], or when a suitable draw solution (DS) stream is available that can be used for concentrating the feed solution (FS) [6,7,8].

In order to successfully apply FO on a larger scale, understanding the process with its influencing and disturbing factors is necessary. This allows subsequent production-scale plants to be designed from the results of smaller laboratory-scale tests. For this upscaling, simulation models are created in the engineering sciences, for the purpose of representing the real process as accurately as possible.

For the FO simulation, the thermodynamic knowledge of existing solutions and the knowledge of mass transport through the membrane of the FO are necessary. There are two types of principle models, namely, computational fluid dynamics (CFD) [9] and simplified models [10]. The authors selected the simplified model due to its ease of adaptability and extension, as well as its potential for effortless transfer to industrial applications. The basics of the simplified model are explained in detail in Section 2.

A given problem of real processes with real solutions Is that the composition of one or more of the present solutions is either partially known or not known at all; thus, no accurate thermodynamic description can be generated for the simulation model.

One approach would be to measure the concentrations of those components of the initial solutions that are expected to have the greatest effect on the thermodynamic properties. However, there is a risk that some substances remain undetected, which has a non-negligible effect on the properties of the entire solution.

Even if all constituents are known, the thermodynamical description comes with a very high effort. This is because solutions with multiple dissolved constituents are more likely to have molecular interactions that are difficult to predict, especially at high concentrations. Thus, for example, calculations of the osmotic pressure according to Van’t Hoff are inadmissible. For complex models such as those according to Bromley [11] or Pitzer [12], which take the interactions into account, empirical data must be available. Moreover, these complex models are reliable only up to a certain concentration dependent on the substance system [13].

Considering the above-argued problems, the authors chose a fully empirical approach to describe the thermodynamic properties. They used a correlation between osmotic pressure and recovery rate created for the substance system of our concern. This method offers the advantage that the required empirical data can easily be determined since only one sum parameter for different recovery rates must be measured. The recovery rate itself has the advantage of being continuously measurable during the process through flow and level measurements. In addition, the complex calculation from the individual concentrations and interaction parameters can be dispensed with.

In the remainder of the paper, the authors first briefly discuss the basics of osmotic pressure and the calculation of solute transport of the FO. Then, the empirical model’s setup and its integration into the FO solute transport calculation are described. To this end, an experiment is carried out to validate the simulation and evaluate the quality of the chosen approach.

## 2. Fundamentals

### 2.1. Osmotic Pressure and Osmotic Coefficient

In order to correctly determine mass transfer, the calculation of osmotic pressures is indispensable. Assuming ideal conditions, the osmotic pressure Π in Pa of a simple electrolyte system that consists of a single electrolyte solved and strongly diluted in water can be determined using the Van’t Hoff equation:

(1) Π=icRT,

where i indicates the Van’t Hoff factor, which considers the stoichiometric dissociation, as well as the degree of dissociation, of a single electrolyte in solution. The molar concentration of the associated electrolyte c is given in mol·m^−3^ and is multiplied by the ideal molar gas constant R in J·mol^−1^·K^−1^, as well as the temperature T in K.

**Figure 1:**
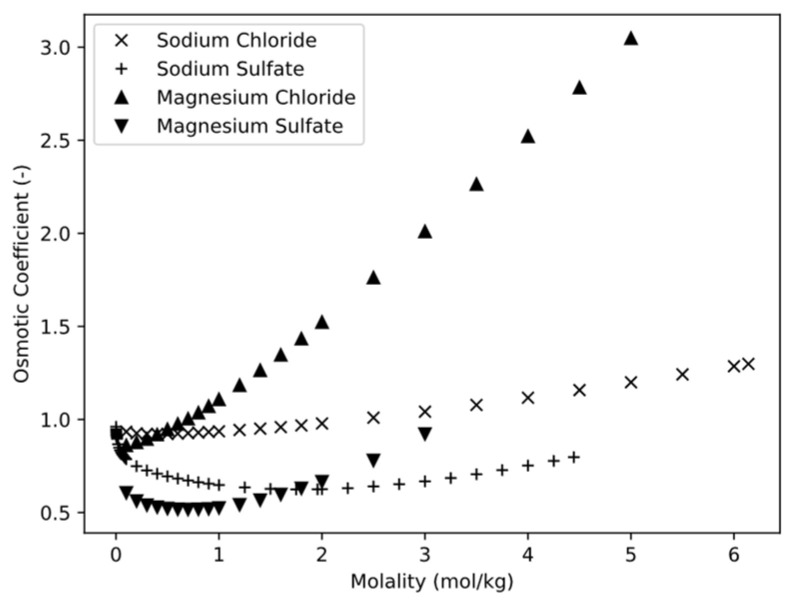
Osmotic pressure coefficient of different electrolyte solutions as a function of molality [15,16,17].

Interactions between ions of equal and opposite charge become bigger with higher concentration, which leads to a deviation from the real behavior of the electrolyte solution. [14]. Especially for the DS, the described deviation increases very strongly depending on the electrolyte used. The relationship between real and ideal behavior is represented by the osmotic coefficient ϕ, which can be calculated according to the following equation [13]:

(2) $$\phi =\frac{-\ln\left(a_{W}\right)}{{\mathrm{vmM}}_{W}}=\frac{c_{\mathrm{osm}}}{c},$$

where $a_{W}$ represents the activity of the solvent, $v_{i}$ is the stoichiometric coefficient of the dissociation reaction of salt i, $m_{i}$ in kg is the mass of the salt, and $M_{W}$ in kg·mol^−1^ is the molar mass of the water. The equation and, thus, the osmotic coefficient can be further interpreted as the ratio of the osmotically effective concentration $c_{\mathrm{osm}}$ in Osmol·m^−3^ and the actual solute concentration c in mol·m^−3^. As can be seen in Figure 1, the osmotic coefficient of sodium chloride is about ϕ = 1 over a wide range of concentrations; therefore, it is legitimate to opt for the Van’t Hoff equation for its simplicity and assess a small error of calculation. Electrolytes such as magnesium chloride and magnesium sulfate, for example, have osmotic coefficients that differ significantly from 1 (see Figure 1). In this case, the application of the Van’t Hoff equation would lead to relative errors of up to 50% for magnesium sulfate or over 100% for magnesium chloride since their osmotic coefficients are less than ϕ = 0.5 or greater than ϕ = 2, respectively.

Several models are available for calculating activity coefficients and water activity in highly concentrated electrolyte solutions, each with their own advantages and disadvantages. The Debye–Hückel model [14] is a simple model that assumes only electrostatic interactions between ions in solution, but it becomes inaccurate at higher concentrations where ion–ion interactions become important. The extended Debye–Hückel model (e.g., the Bromley equation [11]) takes into account ion–ion interactions and solvent structure effects, in addition to electrostatic interactions, making it suitable for higher electrolyte concentrations and more accurate, but also more complex. The Pitzer model [12] is an extension of the Debye–Hückel model that includes additional terms to account for ion–ion interactions and solvent structure effects, allowing it to be used for a wider range of electrolyte concentrations and more complex systems. The NRTL model [18] is a non-electrostatic model that accounts for ion–solvent interactions by considering the nonideal behavior of the solvent and can be used for electrolyte and non-electrolyte solutions. It is the most accurate but also the most complex and computationally intensive model, making it suitable for certain systems where higher accuracy is needed. Ultimately, the choice of which model to use depends on the specific system being studied and the desired level of accuracy, with the Debye–Hückel model being a good starting point for low concentrations of electrolytes, and the extended Debye–Hückel model, Pitzer model, or NRTL model being used for higher concentrations and more complex systems.

It is important to note that accurate predictions of activity coefficients in electrolyte solutions require knowledge of the concentration of each component in the solution. Without accurate knowledge of the concentrations, the calculated activity coefficients may be significantly different from the actual values, leading to large errors in any subsequent calculations or predictions. Therefore, it is important to ensure that the concentrations of all components in the electrolyte solution are known or accurately measured before using any of these models for activity coefficient calculations.

### 2.2. Forward Osmosis and Water Transport

Technical osmosis or forward osmosis is a process in which a solvent—usually water—diffuses from a solution with low osmotic pressure, i.e., the FS, through a semipermeable membrane into a solution with higher osmotic pressure, i.e., the DS. The membrane, consisting of a semipermeable active layer (AL) and a porous support layer (SL) [19], lets the water pass through while retaining other substances, such as salt or organic molecules. This process is self-sustaining until the chemical potentials of the two solutions are equalized. The DS with a high osmotic pressure becomes diluted with the permeate and, thus, reduces the osmotic pressure. The FS is continuously concentrated, having a higher solute concentration and, thus, higher osmotic pressure.

The membrane can be seen as a one-dimensional path within the module, where each point defines a location with different solution properties and, therefore, different material transport.

**Figure 2:**
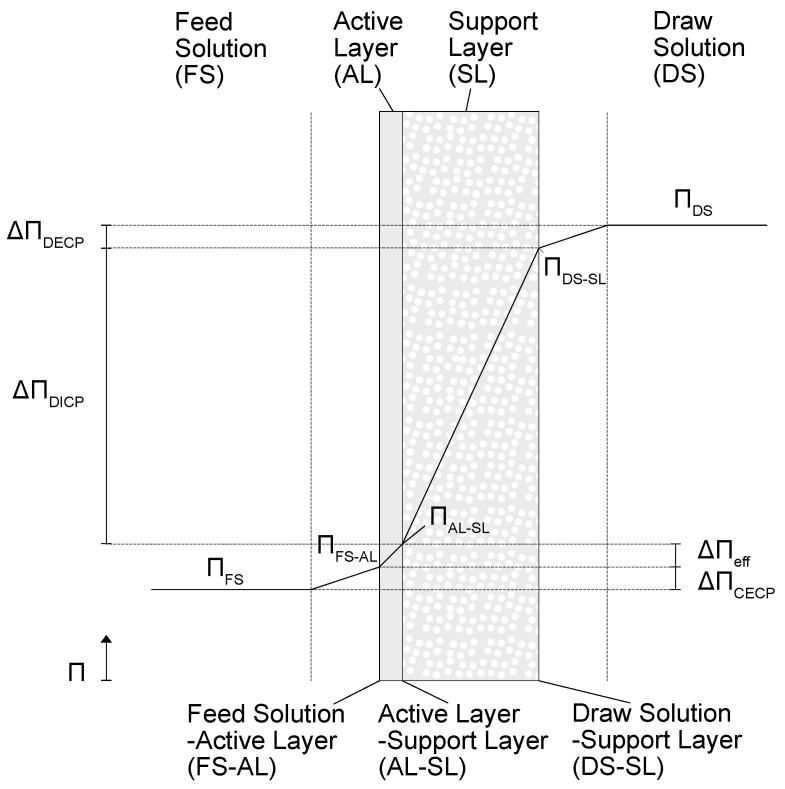
Scheme of material transport for a given membrane location [24,25].

One approach to calculating the material transport for a given location is the solution diffusion model (SDM) [20]. In this simulation, the SDM is used considering the AL–FS membrane orientation, where the active layer of the membrane is in direct contact with FS [21], as shown in Figure 2. This is due to the fact that AL–FS is the most common membrane orientation for commercially available FO modules [22,23].

With the assumption of having similar hydraulic pressures, the driving potential for the material transport in FO is the difference between the osmotic pressures $\Pi_{\mathrm{FS}}$ and $\Pi_{\mathrm{DS}}$. However, as in all membrane processes, so-called concentration polarization (CP) occurs in FO, caused by several concentrations and diluting phenomena, which lead to a loss of the driving potential [26], since molar concentration and osmotic pressure are directly linked to one another (see Section 2.1).

CP can be subdivided into external concentration polarization (ECP), where a concentration gradient occurs in the laminar boundary layer between the bulk stream and the membrane [27], and internal concentration polarization (ICP), where a concentration difference occurs within the porous support layer [13].

ECP on the feed side is caused by solutes from FS that are rejected due to the semipermeable properties of the membrane, thus accumulating immediately in the boundary layer. As a result, the concentration and, therefore, the osmotic pressure $\Pi_{\mathrm{FS}-\mathrm{AL}}$ at the feed solution–active layer interface (FS–AL) is higher than in the feed solution itself. ECP does also appear on the DS side, where the water passes from the membrane into the bulk stream of the DS. Since the permeate has a lower concentration, there is a dilution of the DS at the interface between the DS and the support layer (DS–SL). Therefore, the concentration and osmotic pressure of the DS are reduced to $\Pi_{\mathrm{DS}-\mathrm{SL}}$ toward the membrane.

ICP within the support layer is caused by two opposing mass transfer phenomena. On the one hand, low-concentration permeate flows from the AL through the SL toward the material flow of the DS. On the other hand, an unwanted reverse solute flux (RSF) takes place, where solutes from the DS diffuse through the permeate against the concentration gradient to the boundary layer between AL and SL (AL–SL) [28]. This results in the actual osmotic pressure of the DS, already reduced by ECP, being additionally reduced within the support layer by dilution with permeate to $\Pi_{\mathrm{AL}-\mathrm{SL}}$.

For asymmetric membranes, ICP can reduce the osmotic pressure difference of the bulk streams by 45% to 60%, while ECP can account for 30–40% reduction [29]. Although the extent of ICP and ECP depends on the process conditions and membrane properties, it can be concluded that ICP is the major contributor to the loss of driving force.

Since the osmotic pressure difference ${\Delta \Pi }_{\mathrm{eff}}$ immediately at the boundary layers of the active layer are crucial for the driving force and, therefore, the water flux, the preceding explanation shows that CP is a critical factor in reducing the membrane performance and must strictly be considered in the calculation of the membrane process.

## 3. Materials and Methods

In this study, a simulation of the FO process is presented, where the results of the estimated water flux and the recovery rate are compared with the measured values of laboratory-scale experiments.

### 3.1. Simulation of a Batch Concentration Process

The simulation of the FO process is divided into four parts. First, the osmotic pressure of an unknown solution is calculated on the basis of measurements of the FS for different concentrations and compared to two known electrolyte solutions (see Section 3.1.1). This osmotic pressure is used as a function of the recovery rate to calculate the water flux and recovery rate for a specific location on the membrane path with the solution diffusion model (see Section 3.1.2). Then, the latter calculation is used to numerically estimate the water flux and recovery rate of the entire membrane module (Section 3.1.3) and to predict the change in volume and concentration over time of the batch experiment (Section 3.1.4), which is conducted to verify the simulation.

#### 3.1.1. Osmotic Pressure

A possible solution approach for the simulation of water permeation rates in FO for FS with unknown substance composition could be a fit function of the feed’s osmotic pressure $\Pi_{\left(\mathrm{RR}\right)}$ versus the recovery rate RR (as given in Equation (3)). To do this, a concentration series would have to be prepared previously with the unknown solution using a vacuum rotary evaporator or FO on a laboratory scale. Using cryoscopy or ebullioscopy (see Section 3.2.1), the activity and, from this, the osmotic pressure can then be determined in relation to the concentration. The coefficients $x_{1}$. and x_2_ in Pa can be determined via a GRG nonlinear solver method integrated into Excel.

(3) $$\Pi_{\left(\mathrm{RR}\right)}=\Pi_{0}+\frac{x_{1}\mathrm{RR}+x_{2}{\mathrm{RR}}^{2}}{1-\mathrm{RR}},$$

The advantage of the equation is that the actual concentration of a solution does not have to be known during the concentration process, as it must be the case for usual calculation methods (e.g., according to Van’t Hoff) or more complex activity models (e.g., Pitzer [12] or Bromley [11]). It is sufficient to measure the osmotic pressure at the beginning, to determine the coefficients to assign an osmotic pressure to a solution at each point of the recovery, and to calculate a water flux thereupon. This allows the modeling of solutions where the composition is unknown.

The water recovery or recovery rate RR is calculated using an initial volume with index 0 and the removed volume. The recovery rate can then be related to an input volume flow $Q_{\mathrm{FS},0}$ in m^3^·s^−1^ in a membrane module where the volume $Q_{\mathrm{FS}}$ in m^3^·s^−1^ is the current volume flow rate for a location of a one-dimensional membrane path, as can be seen in Equation (4).

(4) $$\mathrm{RR}=\frac{Q_{\mathrm{FS},0}-Q_{\mathrm{FS}}}{Q_{\mathrm{FS},0}}.$$

The recovery rate can also be determined for a tank in which the feed solution is recirculated in batch mode, i.e., the volume change is equal to the permeate flow. Here, the recovery rate can also be calculated from the initial volume $V_{B,0}$ in m^3^ and the current volume $V_{B}$ in m^3^, as seen in Equation (5).

(5) $$\mathrm{RR}=\frac{V_{B,0}-V_{B}}{V_{B,0}}.$$

#### 3.1.2. Membrane Performance for a Given Location

A common way to calculate the solvent transport of water $J_{W}$ in m^3^·s^−1^·m^−2^ as described by SDM is to use the permeability A in m^3^·s^−1^·m^−2^·Pa^−1^, hydraulic pressure difference Δp in Pa, and osmotic pressure difference ΔΠ in Pa. Solvent transport occurs between feed solution membrane interface and active layer–support layer interface which is shown in Figure 1 and described in Equation (6) [2].

(6) $$J_{W}=A\left(\Delta \Pi -\Delta p\right)=A\left(\left(\Pi_{\mathrm{DS},\mathrm{BL}}-\Pi_{\mathrm{FS},\mathrm{BL}}\right)-\left(p_{\mathrm{DS},\mathrm{BL}}-p_{\mathrm{FS},\mathrm{BL}}\right)\right).$$

The specific salt flow $J_{S}$ mol·s^−1^·m^−2^ is calculated in Equation (7) using the salt permeability B in m^3^·s^−1^·m^−2^ and the present concentration difference at the boundary layer $c_{\mathrm{FS}-\mathrm{AL}}$ and $c_{\mathrm{DS}-\mathrm{AL}}$ of the active layer and corresponding solutions [30].

(7) $$J_{S}=B∆c=B\left(c_{\mathrm{FS}-\mathrm{AL}}-c_{\mathrm{DS}-\mathrm{AL}}\right).$$

In order to specify the concentration of the unknown FS that is derived from the previously determined fit function in Equation (3) of the osmotic pressure, Equation (8) is used.

(8) $$C_{\mathrm{osm}}=\frac{\Pi_{\left(\mathrm{RR}\right)}}{\mathrm{RT}}.$$

Furthermore, this assumption limits the fitting function to concentration ranges where the constituents are completely dissolved. If any components were to precipitate, they would no longer contribute to the osmotic pressure. In the solution diffusion model, the external concentration polarization is described according to Equation (9), where the molar concentrations at the boundary between the feed solution and active layer $C_{\mathrm{FS}-\mathrm{AL}}$, as well as of the bulk stream of the FS $C_{\mathrm{FS}}$, are required [31]. The transport coefficient $k_{\mathrm{FS}}$ in m·s^−1^ indicates the solute diffusion through the laminar interface.

(9) $$\frac{\left(c_{\mathrm{FS}-\mathrm{AL}\;}+\frac{J_{W}}{J_{S}}\right)}{\left(c_{\mathrm{FS}\;}+\frac{J_{W}}{J_{S}}\right)}=\exp\left(\frac{J_{W}}{k_{\mathrm{FS}}}\right).$$

(10) $$\frac{\left(c_{\mathrm{FS}-\mathrm{AL}\;}+\frac{J_{W}}{J_{S}}\right)}{\left(\frac{\Pi_{\mathrm{FS},\left(\mathrm{RR}\right)}}{\mathrm{RT}}+\frac{J_{W}}{J_{S}}\right)}=\exp\left(\frac{J_{W}}{k_{\mathrm{FS}}}\right).$$

The molar concentration of the FS bulk stream c_FS_ in Equation (9) can now be substituted by Equation (8) to obtain Equation (10), which describes the calculation of the external concentration polarization (ECP) on the feed side.

Equations (11) and (12) for calculating the external dilute concentration polarization (DECP) [31], as well as those for the internal concentration polarization (ICP) [32], are used without substitution since the concentration of the initial DS, which can be freely selected by the user in most cases, is known. It should be neglected here that dissolved substances can also diffuse from the feed into the DS.

(11) $$\frac{\left(C_{\mathrm{DS}-\mathrm{SL}\;}+\frac{J_{W}}{J_{S}}\right)}{\left(C_{\mathrm{DS}\;}+\frac{J_{W}}{J_{S}}\right)}=\exp\left(-\frac{J_{W}}{k_{m}}\right).$$

(12) $$\frac{\left(C_{\mathrm{AL}-\mathrm{SL}\;}+\frac{J_{W}}{J_{S}}\right)}{\left(C_{\mathrm{DS}-\mathrm{SL}\;}+\frac{J_{W}}{J_{S}}\right)}=\exp\left(-\frac{J_{W}}{k_{\mathrm{DS}}}\right).$$

The mass transfer coefficients $k_{\mathrm{FS}}$ and $k_{\mathrm{DS}}$ of the laminar boundary layers are calculated according to the film theory for flow processes, shown in Equation (13). For this purpose, the material-specific diffusivity $D_{S}$ in m^2^·s^−1^ is multiplied by the dimensionless Sherwood number Sh and divided by the hydraulic diameter $d_{h}$ in m, using the fiber lumen inner diameter and space between the lumen shell and module shell for FS and DS, respectively [33].

(13) $$k_{\mathrm{FS}}=k_{\mathrm{DS}}=\frac{D_{S}\mathrm{Sh}}{d_{h}}.$$

The Sherwood number (Equation (14)) is calculated using the Reynolds number Re and the Schmidt number Sc [34]. The coefficient α and the exponents β and γ differ according to flow geometry and are determined experimentally.

(14) Sh=αReβScγ.

The Reynolds number Re (Equation (15)) is in turn determined with the flow velocity u in m·s^−1^ on the feed or draw side and the kinematic viscosity, which is considered constant for this simulation [34].

(15) Re=udhv.

The Schmid number Sc (Equation (16)), which is needed to calculate the Sherwood number, is in turn calculated from diffusivity and kinematic viscosity [34].

(16) $$\mathrm{Sc}=\frac{v}{D_{S}}.$$

The mass transfer coefficient of the support layer $k_{M}$ in m·s^−1^ is determined from the diffusivity and the structure parameter S in m [33]. The latter can be calculated theoretically from the porosity, the tortuosity, and the thickness of the membrane [35]. Practically, methods to determine the tortuosity are lacking, which is why the structure parameter S is determined experimentally.

(17) $$k_{M}=\frac{D_{S}}{S}.$$

Lastly, the diffusivity $D_{s}$ is determined using Equation (18) [36].

(18) $$D_{s}=\left[\left(\frac{8e^{-7}C_{\mathrm{NaCl}}^{2}}{M_{\mathrm{NaCl}}i_{\mathrm{NaCl}}}\right)-\left(\frac{0.0004C_{\mathrm{NaCl}}}{M_{\mathrm{NaCl}}i_{\mathrm{NaCl}}}\right)+1.5198\right]\times 10^{-9}.$$

#### 3.1.3. Overall Module Performance

**Figure 3:**
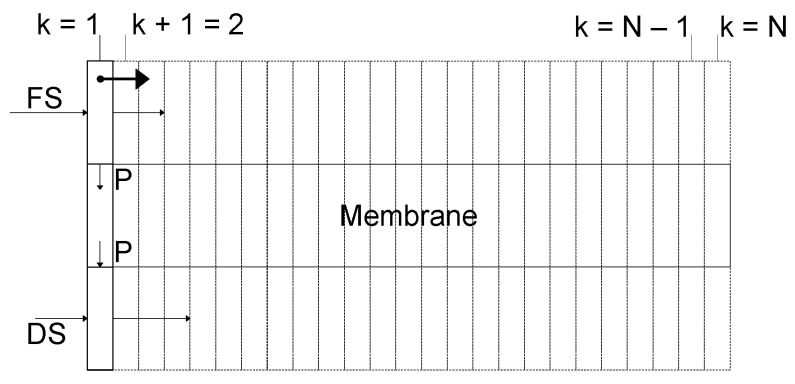
Scheme of material transport for a given membrane cross-section.

Equations (1)–(18) can be used to calculate volume flow rates Q in m^3^·s^−1^ and the osmotic substance flow rates N in Osmol·s^−1^ for a segment k in a one-dimensional membrane path. The whole membrane consists of N = 25 segments, as shown in Figure 3. In this process, a balance for the FS and DS in a co-current flow direction is created, shifting over the entire length of the membrane module with equal step sizes.

The calculation of the water balance for the FS and DS for a segment k can be seen in Equations (19) and (20), respectively. For each new segment k+1 with the membrane area $\frac{A_{m}}{N}$ in m^2^, a permeate flow $Q_{P,k+1}$ in m^3^·s^−1^ is calculated, which is subtracted from the FS and added to the DS.

(19) $$Q_{\mathrm{FS},k+1}=Q_{\mathrm{FS},k}-Q_{P,k+1}=Q_{\mathrm{FS},k}-\frac{A_{m}}{N}J_{W,k+1}.$$

(20) $$Q_{\mathrm{DS},k+1}=Q_{\mathrm{DS},k}+Q_{P,k+1}=Q_{\mathrm{DS},k}+\frac{A_{m}}{N}J_{S,k+1}.$$

The equations for the output flow rates (Equations (21) and (22)) can then be derived. Here, numerical integration is performed over the length of the membrane surface for all N segments present. The increase in volume of the DS or decrease in volume of the FS in relation to the complete module is, therefore, due to the observable permeate flow $Q_{P,\mathrm{obs}}$.

(21) $$Q_{\mathrm{FS},\mathrm{out}}=Q_{\mathrm{FS},\mathrm{in}}-Q_{P,\mathrm{obs}}=Q_{\mathrm{FS},\mathrm{in}}-\frac{A_{m}}{N}\overset{N}{\underset{k=1}{\sum }}J_{W,k}.$$

(22) $$Q_{\mathrm{DS},\mathrm{out}}=Q_{\mathrm{DS},\mathrm{in}}+Q_{P,\mathrm{obs}}=Q_{\mathrm{DS},\mathrm{in}}+\frac{A_{m}}{N}\overset{N}{\underset{k=1}{\sum }}J_{W,k}.$$

The permeate flow characterizing the process can, therefore, be determined from the difference between the respective input and output flows using Equation (23).

(23) $$Q_{P,\mathrm{obs}}=Q_{\mathrm{FS},\mathrm{in}}-Q_{\mathrm{FS},\mathrm{out}}=Q_{\mathrm{DS},\mathrm{out}}-Q_{\mathrm{DS},\mathrm{in}}.$$

The balances of the salt streams $N_{\mathrm{FS}}$ (Equation (24)) and $N_{\mathrm{DS}}$ (Equation (25)) in this simulation do not refer to the actual amount of substance present, but only to the osmotically effective amount of substance. The balances are calculated in the same way and time as the volume flows. The DS consists of an initial salt stream, which is reduced only by undesired RSF during the concentration process. The calculated osmotically effective substance flow rate for the FS, thus, consists of the fraction of salt reflux from the DS, which is added in each segment to the fraction of salt already contained due to RSF. Furthermore, the already present osmotically effective substance flow rate is recalculated for each segment by means of the volume flow rate $Q_{\mathrm{FS}}$ and osmolal concentration $C_{\mathrm{FS},\mathrm{osm}}$ according to the present recovery rate (Equation (8)).

(24) $$N_{\mathrm{FS},k+1}=N_{\mathrm{FS},k}+N_{P,k+1}={\left(Q_{\mathrm{FS}}C_{\mathrm{FS},\mathrm{osm}\left(\mathrm{RR}\right)}\right)}_{k+1}+\frac{A_{M}}{N}J_{S,k+1}.$$

(25) $$N_{\mathrm{DS},k+1}=N_{\mathrm{DS},k}-N_{P,k+1}=N_{\mathrm{DS},k}-\frac{A_{M}}{N}J_{S,k+1}.$$

If the material flows are integrated over all segments N, the substance flows at the outputs can be represented by Equations (26) and (27).

(26) $$N_{\mathrm{FS},\mathrm{out}}={\left(Q_{\mathrm{FS}}C_{\mathrm{FS},\mathrm{osm}}\right)}_{k}+\frac{A_{M}}{N}\overset{N}{\underset{k=1}{\sum }}J_{S,k}.$$

(27) $$N_{\mathrm{DS},\mathrm{out}}=N_{\mathrm{DS},\mathrm{in}}-\frac{A_{M}}{N}\overset{N}{\underset{k=1}{\sum }}J_{S,k}.$$

The salt stream flow caused by RSF, through the membrane, can be determined from the mass flow difference of the DS using Equation (28).

(28) $$N_{P,\mathrm{obs}}=N_{\mathrm{DS},\mathrm{in}}-N_{\mathrm{DS},\mathrm{out}}.$$

#### 3.1.4. Batch Tank Balances

For the concentration of a storage tank B in batch mode, as carried out in the experiment, balances are shown using Equation (29) as a function of time t in s. The timestep size in the simulation is selected as Δt=1 s. Thus, the volume $V_{B}$ of the feed storage tank changes due to the previously calculated permeate flow $Q_{p}$.

(29) $$V_{B\left(t\right)}=V_{B\left(t-\Delta t\right)}-{\Delta \mathrm{tQ}}_{P\left(t-\Delta t\right)}.$$

The osmotic concentration in the feed tank $C_{B,\mathrm{osm}\left(t\right)}$ is calculated, as shown in Equation (30), analogous to the material flow within the module, taking into account the correlation of the osmotic pressure as a function of the recovery rate, partly from the amount of substance that additionally enters the tank as RSF per time interval.

(30) $$C_{B,\mathrm{osm}\left(t\right)}=\frac{\Pi_{B\left(\mathrm{RR}\right)}}{\mathrm{RT}}+\frac{N_{B,\mathrm{RSF}\left(t-\Delta t\right)}+{\Delta \mathrm{tN}}_{P,\mathrm{obs}\left(t-\Delta t\right)}}{V_{B\left(t\right)}}.$$

Lastly, a specific water flux $J_{W,B\left(t\right)}$ can also be obtained using Equation (31) for the circulating FS in the storage tank by dividing the observed permeate flux per time interval $Q_{P,\mathrm{obs}\left(t\right)}$ by the membrane area $A_{M}$ in m^2^.

(31) $$J_{W,B\left(t\right)}=\frac{Q_{P,\mathrm{obs}\left(t\right)}}{A_{M}}.$$

Table 1 shows a summary of the initially used parameters for the calculation. In this calculation, the water permeability, salt permeability, and Sherwood parameters from a study conducted by Munubarthi [35] for the Aquaporin hollow fiber modules HFFO2 are used.

### 3.2. Experimental

#### 3.2.1. Setup

**Figure 4:**
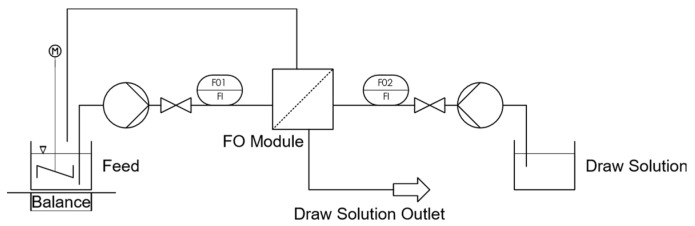
Experimental setup.

The experimental test setup (Figure 4) for the validation of the described simulation consisted of two tanks containing the FS and the DS. The solutions were transported from the tanks via a centrifugal pump (NEMP50/7) from Harton, Germany to the respective inlets of the hollow fiber FO module (HFFO2) from Aquaporin, Denmark, through which they flowed in direct current (the properties of the module can also be seen in Table 1). Here, the feed entered the lumen of the hollow fiber module, and contacted the active layer, creating the AL–FS membrane orientation. The DS flowed on the shell side of the support layer. To achieve the best possible degassing, the module was installed vertically in the test rig and flowed through in an upstream direction. The DS was stored in a separate tank, whereas the FS was fed back into the supply tank. Here, an electric table stirrer ensured optimum mixing of the FS and the FS concentrate flowing back. The mass of the feed tank was measured using a sartorius laboratory precision scale in order to determine the permeate flow via mass reduction. Control valves installed directly after the pumps enabled adjusting the flow rates manually. The flow rates could be measured using GEMÜ float flow meters for an initial estimate.

Since the change in mass in the feed tank was particularly high at the beginning, a video camera was used to read the value of the balance simultaneously with a common laboratory stopwatch.

Magnesium chloride and magnesium sulfate in technical grade from manufacturers Deusa (Bleicherode, Germany) and K+S (Kassel, Germany) were used. The Osmomat 3000 cryoscope from Gonotec (Berlin, Germany) was used to check the osmotically effective concentration. It internally converts the cryoscopic constant of the water from the measured freezing point of the sample volume to osmolality. This allows, firstly, verifying the concentration of the prepared solutions and, secondly, adjusting osmotic concentrations as initial input for the simulation.

#### 3.2.2. Test Execution

Before the main experiment can start, the membrane must be sufficiently wetted with water and brought to the correct temperature, as this has a significant effect on the diffusion and, thus, membrane performance. For this purpose, a rinsing procedure for both cycles was conducted, where DI water was run at T = 25 °C for 30 min in batch mode. In order to compare the measured values with the standard Aquaporin test for the determination of water flux [23], the same flow rates of 60 L/h for the FS and 25 L/h for the DS were chosen. Using a measuring cylinder and a stopwatch, the true volume flow rates were measured under triple repetitions, which in turn served as initial values for the subsequent simulation.

After rinsing the cycles with DI water, a standard test according to Aquaporin [23] was performed to verify the actual performance of the applied membrane module. For this, DI water was used as the FS and a 0.5 M NaCl solution was used as the DS. Temperature and volumetric flow rates were the same as for rinsing with water. If deviations of the permeate flux and, thus, the water permeability A from the manufacturer’s specifications were noticed during this test, they were taken into account later when validating the calculation.

The tests with the electrolyte solutions were each carried out directly after a 30 min rinse with water so that any ions present from the preliminary test were removed, and the module and the tubing were filled with water; thus, the same starting conditions applied for each test conducted. The FS was prepared with DI water and magnesium chloride or magnesium sulfate to obtain 0.2 M solutions at V = 5 L. This low initial concentration allowed the largest possible range of concentration to be achieved during the concentration process. The DS was prepared in sufficient quantity as a 1 M NaCl solution. At the beginning, as well as every further 60 s, a 50 µL sample of the feed was taken and analyzed in the cryoscope to estimate the osmotic concentration. The measured value was compared with the calculated one.

For comparison, the measured permeate flux J_W_ of the tank was calculated from the volume decrease Δ$V_{B}$ for time span Δt between two measuring points, as shown in Equation (32).

(32) $$J_{W}=\frac{{\Delta V}_{B}}{\Delta t}.$$

The change in volume in the storage tank was calculated, as shown in Equation (33), from the measured mass difference Δm divided by the density ρ in kg·m^−3^.

(33) $${\Delta V}_{B}=\frac{\Delta m}{\rho }.$$

The osmotic pressure Π was calculated (see Equation (34)) from the water density $\rho_{W}$ in kg·m^−3^, the ideal gas constant R, and the temperature T, together with the osmolality $b_{\mathrm{osm}}$, measured by cryoscopy.

(34) $$\Pi =b_{\mathrm{osm}}\rho_{W}\mathrm{RT}.$$

## 4. Results and Discussion

### 4.1. Standard Test

A simulation according to the standard test of the membrane manufacturer was carried out to measure the water flux of the present membrane and to compare the simulation results using the initial parameter set of Munubarthi [35], enhanced with the fit function for osmotic pressure.

Since this section of the study is aimed at taking a closer look at water transport and its related variable, salt transport is not considered in comparison with the standard test. The results are shown in Table 2. The Aquaporin HFFO2 membrane has a water flux of J_w_ = 11.5 ± 1.5 L·m^−2^·h^−1^ (LMH) and a recovery rate of RR = 0.42 when a 0.5 M sodium chloride solution at 25 L/h and DI water at 60 L/h are passed through the module in single-pass mode. A calculation with the permeabilities according to Munubarthi [35] showed a slight underperformance with a flux of 9.6 LMH and a recovery of RR = 0.35 when passing the module once. This could be due to the fact that Munubarthi [35] used a highly concentrated solution of 100 g/L assuming an ideal osmotic pressure according to the Van’t Hoff equation, and considering that the coefficient for the water permeability was calculated too low.

By adjusting the membrane parameters A and B, it was possible to calculate the same water flux and RSF mentioned in the Aquaporin datasheet for its HFFO2 module [23]. These adjusted parameters, which are shown in Table 2, were used for further simulations.

### 4.2. Osmotic Pressure Correlation

The samples were analyzed using the osmometer, and their osmotically effective solute concentration was determined, converted to a real osmotic pressure using the Van’t Hoff equation, and plotted as a function of the recovery rate. Since there were problems during the startup of the experiment to reach a stable equilibrium state, the recovery rate was set to RR = 0 for t = 2 min. Accordingly, the osmotic pressure measured at this time was considered to be Π_0_. The x_1_ and x_2_ parameters of Equation (3) were estimated using the GRG nonlinear solver method integrated into Excel. Here, the relative error between the fit function and the measured value was as small as possible. The calculated parameters of Equation (3) for the FS used in both experiments are shown in Table 3.

**Figure 5:**
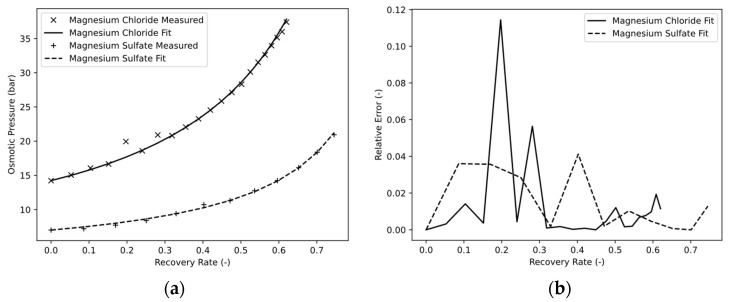
Osmotic pressure (**a**) and relative error (**b**) as a function of the recovery rate.

Figure 5a shows the osmotic pressure as a function of the recovery rate. Measured values are compared with the fit function. It is shown that the chosen mathematical function gave a good approximation of the measured values. This was, among other things, due to the fractional rational property, whereby the fit function converged to infinity at recovery rates of 1. An advantage is that Π_0_ is directly determined correctly, and the function is developed starting from the true value, whereby small deviations are found, particularly with low recovery rates. In addition, Π_0_ with an unknown solution only corresponds to the osmotic pressure, which the FS has in the initial state. This is often determined during initial analyses and would not need an additional measurement for the empirical determination.

As expected, magnesium chloride and magnesium sulfate showed strongly differing osmotic pressures. Both salts are strong electrolytes, with magnesium chloride dissociating into 50% more ions during the dissociation reaction with a stoichiometric coefficient of v = 3 than magnesium sulfate with a stoichiometric coefficient of v = 2. Hence, magnesium chloride should show a 50% higher osmotic pressure compared to magnesium sulfate under ideal assumptions. The fact that these assumptions do not apply was explained above (Section 3.1.1) with the osmotic factor (see Figure 1) and was confirmed by the experiment. It is shown that the osmotic pressure of the FS (see Figure 5a) increased during the concentration process. This was due to the water removal, which led to a concentration of the osmotic pressure producing solutes. The increase in osmotic pressure of magnesium chloride was stronger than that of magnesium sulfate because of the already mentioned higher dissociation coefficient and because of the osmotic coefficient [15,16,17].

The strong incline of magnesium chloride is also shown by parameter x_1_ = 13.71 of the linear term, in contrast to only x_1_ = 4.85 for magnesium sulfate. It is also worth noting that the latter salt, with x_2_ = 0, has no quadratic term, and the fit function can be constructed by only two measurement points to be mathematically determined.

Figure 5b shows the relative error of the fit function to the measured values. For magnesium chloride, two flawed measured points can be seen at approximately RR = 0.2 and RR = 0.29, which were probably due to sampling errors. If excluded, the fit functions for both solutions showed a relative error of less than 5% for the entire concentration process.

### 4.3. Experiments and Simulations

**Figure 6:**
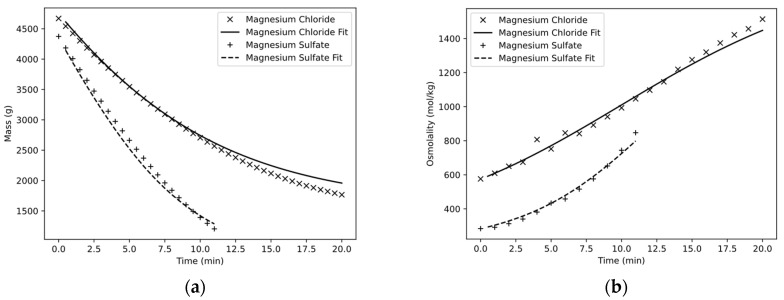
Mass (**a**) and osmolality (**b**) of the storage tank as a function of time.

The experiments were carried out with magnesium chloride and magnesium sulfate. Figure 6a shows that the two solutions already had different masses after the startup time of 2 min, although they were both prepared with a total weight of 5 kg. This was due to the fact that magnesium chloride, with a higher osmotic pressure, had a lower driving potential at the beginning, which in turn led to increased water flux and, thus, a decrease in volume and mass. The test with magnesium sulfate was terminated after only 11 min, as a sufficient concentration was achieved. The test with magnesium chloride was terminated after 20 min, as a sufficient recovery rate was achieved. The measured data show a steady behavior and do not seem to contain any noticeable deviations. The simulation seemed to successfully describe the mass decreases. Whereas, for the magnesium sulfate solution, a stronger mass decrease was predicted during the first minutes than was actually measured, the curves converged again toward the end of the experiment such that the masses exhibited congruence. For the magnesium chloride solution, on the other hand, the mass during the batch concentration could be predicted almost precisely up to 8 min until the simulation finally underperformed, and the mass of the receiver tank after 20 min showed a difference of 192 g (1958 g vs. 1766 g), corresponding to a relative error of 10.8%.

Figure 6b shows the osmometric measurements of osmolality as a function of the experimental time. Here, the two solutions also showed a different pattern. In the case of magnesium chloride, the osmolality increased quasi-linearly from 576 mOsmol/kg to 1514 mOsmol/kg, corresponding to a factor of 2.63. In the case of magnesium sulfate, on the other hand, a curve characteristic of the concentration process can be seen, in which the osmolality increased particularly rapidly from an initial 284 mOsmol/kg toward the end of the experiment to 847 mOsmol/kg by a factor of 2.98. While the simulation was able to predict the progression relatively accurately for both cases, the osmolality for magnesium chloride was underestimated by 67 mOsmol/kg toward the end of the experiment at 1447 mOsmol/kg, corresponding to a relative error of 4.4%.

One of the most important values for the performance evaluation of the simulation and the process of FO is the water flux, which indicates the permeate flow in relation to the membrane area. Thus, it is primarily used for comparisons between different membrane manufacturers or operating parameters, or it serves as an auxiliary value for the upscaling of membrane areas. The water flux measured in the experiment over the experimental procedure can be seen in Figure 5a. Magnesium chloride started at a water flux of 6.61 LMH and then continuously decreased to 1.33 LMH. This could only be partially described well by the simulation. The maximum relative error at the beginning was about 8% and increased to about 20% at t = 15.5 min. Similar behavior is shown for magnesium sulfate, where the initial error of the simulation was also 8% but increased to 30% by the end of the experiment at t = 11 min.

In general, three different explanations were found to have the most significant impact on the difference between the simulation and experimental results:

1. Incorrect diffusion coefficients for osmotic particle diffusion

The diffusion coefficient mentioned in Equations (13) and (16)–(18) refers to the common solute concentration and not to the osmotic effective one. As a result, the diffusion coefficient differs considerably for electrolytes which have an osmotic coefficient greatly different from 1 at the given concentrations. This can be especially seen for magnesium sulfate, which has an osmotic coefficient between 0.5 and 0.6 at the investigated concentrations [17]; there was a greater error over the duration of the experiment than for magnesium chloride, which is estimated to have an osmotic coefficient of only 0.8–1.2 [17] (see Figure 1 (0.2–1 mol/kg)). In addition, it must be taken into account that the diffusivity of unknown solutions can change significantly with the concentration.

2. Accumulation of errors during simulation

In this study, the simulation was designed to run over multiple concentration ranges by batch-driving the feed, thus validating the simulation for just those ranges. Since the simulation, similar to the experiment, was started with an initial set of parameters and was not adjusted to the measured values during the calculation, errors from the beginning of the simulation were dragged through the complete calculation. Thus, even a small deviation in the calculation could have resulted in a large error in the later process.

3. Assumption of the parameters for the simulation

It is conceivable that the parameters adopted by Munubarthi were inappropriate for the simulation of this study. It has already been shown that the water permeability A and the salt permeability B are not suitable, as they were determined under different conditions. It may be that the fit was still not correct under the aspect of the incorrect diffusivities and structure parameters found in this study.

**Figure 7:**
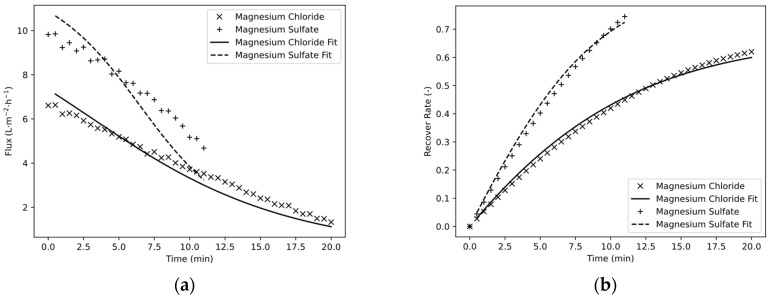
Water flux (**a**) and recovery rate of storage tank (**b**) as a function of time.

Although partly large deviations were previously recorded in the permeate flow, there are nevertheless good agreements for the recovery rate of the storage tank (Figure 7b). This is partly due to the fact that the initial values of the calculation were set at RR = 0, and the subsequent calculation develops from this point on. Furthermore, the water flux could be mathematically derived from the recovery rate, making it more sensitive and prone to deviations between calculated and measured values. Accordingly, the deviation between simulation and measured values was small in the initial range. The magnesium sulfate solution was concentrated to RR = 0.74 within 11 min according to the high permeate flows. In contrast, the magnesium chloride solution was only concentrated to RR = 0.62 within 22 min. The relative error of the calculation toward the end of the test was 2.9% for magnesium sulfate and 3.3% for magnesium chloride.

## 5. Conclusions and Outlook

The substitution of the calculation of the osmotic pressure with a previously determined fit function showed a sufficient result. However, optimizations need to be made in order to calculate water flux more accurately. The diffusion coefficient or diffusivity must always be assumed or determined iteratively. At this stage, this limits the applicability of the simulation. Under the assumption that the permeabilities, together with the remaining membrane parameters, are independent from the given solutions, then the diffusivity correlation should be determined as a function of the recovery rates in order to achieve accurate results.

Furthermore, the calculation is to be extended so that, in addition to an FS, a DS with unknown composition can also be used to calculate the flux. This use case is very important, especially in hybrid systems of FO, where the DS is continuously recovered, as there is a temporal change in the composition of the FS. Under certain circumstances, the performance of the FO can be strongly changed by this. To be able to calculate this behavior during the design process, the chosen approach would be very beneficial to assure the wished concentration performance.

The RSF should be investigated in more detail for the method. For DS of known composition, photometric measurement can be used to calculate the concentration loss. Deriving the low salt loss via conductivity is inappropriate for the DS because small concentration changes cannot be detected reliably in the presence of high-conductivity-producing concentrations.

In this study, the Aquaporin hollow fiber module was used in the simulation to validate the proposed hypothesis. The selection of a commercial module also constituted a number of parameters that influence the process performance, including membrane and flow channel areas, Sherwood parameters, hydraulic diameters, permeability constants of the membrane, and structural constants. These parameters play a crucial role in determining the efficiency and effectiveness of the process.

In the future, the calculation model will be verified for other commercially available modules of the FO. Accordingly, not only will membrane types (TFC and CTA) be varied, but different module geometries will also be tested. Once the validation process is complete, the robustness of the simulation model will be further confirmed using real-world solutions such as digestates from biogas plants.

## Figures and Tables

**Table 1:** Initial simulation parameter.

Parameter	Value	Parameter	Value
$Q_{\mathrm{FS},\mathrm{in}}$	60 L·h^−1^ [23]	$\rho_{W\left(T=25{}^{\circ}C\right)}$	997.04 kg·m^−3^
$C_{\mathrm{FS}\;}$	0.2 mol·l^−1^	$\upsilon_{W}$	0.8926 mm^2^·s^−1^
$Q_{\mathrm{DS},\mathrm{in}}$	25 L·h^−1^ [23]	T	25 °C
$C_{\mathrm{DS}\;}$	1 mol·L^−1^	p	10^5^ Pa
A	0.914 L·m^−2^·h^−1^·bar^−1^ [35]	S	194.79 µm [35]
B	0.34 L·m^−2^·h^−1^ [35]	$A_{M}$	2.3 m^2^ [12]
$d_{h,\mathrm{FS}}$	195 µm *	$l_{M}$	0.27 m [37]
$d_{h,\mathrm{DS}}$	1080 µm *	$A_{Q,\mathrm{FS}}$	426 mm^2^ [35]
$\alpha_{\mathrm{FS}}$	0.0273 [35]	$\alpha_{\mathrm{DS}}$	0.734 [35]
$\beta_{\mathrm{FS}}$	1.416 [35]	$\beta_{\mathrm{DS}}$	0.084 [35]
$\gamma_{\mathrm{FS}}$	0.33 [35]	$\gamma_{\mathrm{DS}}$	0.33 [35]
R	8.314426 J·mol^−1^·K^−1^	$A_{Q,\mathrm{DS}}$	3770 mm^2^ *
Δt	1 s	N	25

* Values calculated from manufacturer information.

**Table 2:** Comparison of standard test and simulation for different sets of parameters.

Test	A (L·m^−2^·h^−1^·bar^−1^)	B (L·m^−2^·h^−1^)	RR_obs_	Flux_obs_ (LMH)
Aquaporin HFFO2 Standard test	0.25	0.019	0.42	11 ± 1.5
Munubarthi [35]	0.914	0.012	0.35	9.6
This study	1.325	0.017	0.42	11.1

**Table 3:** Parameters of the empirical fit function (Equation (3)).

Substance	Π_0_	x_1_	x_2_
Magnesium chloride	14.24 bar	13.71	1.22
Magnesium sulfate	7.02 bar	4.85	0

## Data Availability

Data are contained within the article.

## References

[1] Boretti A., Rosa L. (2019). Reassessing the projections of the World Water Development Report. NPJ Clean Water.

[2] Nassrullah H., Anis S.F., Hashaikeh R., Hilal N. (2020). Energy for desalination: A state-of-the-art review. Desalination.

[3] Qasim M., Badrelzaman M., Darwish N.N., Darwish N.A., Hilal N. (2019). Reverse osmosis desalination: A state-of-the-art review. Desalination.

[4] Suwaileh W., Pathak N., Shon H., Hilal N. (2020). Forward osmosis membranes and processes: A comprehensive review of research trends and future outlook. Desalination.

[5] Kim B., Gwak G., Hong S. (2017). Review on methodology for determining forward osmosis (FO) membrane characteristics: Water permeability (A), solute permeability (B), and structural parameter (S). Desalination.

[6] Su J., Chung T.-S., Helmer B.J., de Wit J.S. (2012). Enhanced double-skinned FO membranes with inner dense layer for wastewater treatment and macromolecule recycle using Sucrose as draw solute. J. Membr. Sci..

[7] Phuntsho S., Shon H.K., Hong S., Lee S., Vigneswaran S. (2011). A novel low energy fertilizer driven forward osmosis desalination for direct fertigation: Evaluating the performance of fertilizer draw solutions. J. Membr. Sci..

[8] Bamaga O., Yokochi A., Zabara B., Babaqi A. (2011). Hybrid FO/RO desalination system: Preliminary assessment of osmotic energy recovery and designs of new FO membrane module configurations. Desalination.

[9] Liang Y., Fletcher D. (2023). Computational fluid dynamics simulation of forward osmosis (FO) membrane systems: Methodology, state of art, challenges and opportunities. Desalination.

[10] Singh S.K., Sharma C., Maiti A. (2023). Modeling and experimental validation of forward osmosis process: Parameters selection, permeate flux prediction, and process optimization. J. Membr. Sci..

[11] Bromley L.A. (1973). Thermodynamic properties of strong electrolytes in aqueous solutions. AIChE J..

[12] Khraisheh M., Dawas N., Nasser M., Al-Marri M., Hussien M.A., Adham S., McKay G. (2019). Osmotic pressure estimation using the Pitzer equation for forward osmosis modelling. Environ. Technol..

[13] Luckas M., Krissmann J. (2001). Thermodynamik der Elektrolytlösungen.

[14] Huckel E., Debye P. (1923). Zur Theorie Der Elektrolyte. I. Gefrierpunktserniedrigung Und Verwandte Erscheinungen. Phys. Z.

[15] Goldberg R.N. (1981). Evaluated activity and osmotic coefficients for aqueous solutions: Thirty-six uni-bivalent electrolytes. J. Phys. Chem. Ref. Data.

[16] Scatchard G., Hamer W.J., Wood S.E. (1938). Isotonic Solutions. I. The Chemical Potential of Water in Aqueous Solutions of Sodium Chloride, Potassium Chloride, Sulfuric Acid, Sucrose, Urea and Glycerol at 25°^1^. J. Am. Chem. Soc..

[17] Robinson R.A., Stokes R.H. (2002). Electrolyte Solutions.

[18] García E., Rodriguez L., Ferro V., Valverde J. (2019). Prediction of multicomponent ION exchange equilibria by using the e-NRTL model for computing the activity coefficients in solution. Fluid Phase Equilibria.

[19] Rufuss D.D.W., Hosseinipour E., Arulvel S., Davies P. (2023). Complete parametric investigation of a forward osmosis process using sodium chloride draw solution. Desalination.

[20] Wijmans J., Baker R. (1995). The solution-diffusion model: A review. J. Membr. Sci..

[21] Zhao S., Zou L., Mulcahy D. (2011). Effects of membrane orientation on process performance in forward osmosis applications. J. Membr. Sci..

[22] TOYOBO Co. LTD. FO Module Brochure. https://www.toyobo-global.com/seihin/ro/brochure/exhibit/.

[23] Aquaporin HFFO2 Data Sheet. https://aquaporin.com/forward-osmosis-membranes/.

[24] Zargar M., Ujihara R., Vogt S.J., Vrouwenvelder J.S., Fridjonsson E.O., Johns M.L. (2020). Imaging of membrane concentration polarization by NaCl using 23Na nuclear magnetic resonance. J. Membr. Sci..

[25] Lin S. (2016). Mass transfer in forward osmosis with hollow fiber membranes. J. Membr. Sci..

[26] Tan C.H., Ng H.Y. (2008). Modified models to predict flux behavior in forward osmosis in consideration of external and internal concentration polarizations. J. Membr. Sci..

[27] Suh C., Lee S. (2013). Modeling reverse draw solute flux in forward osmosis with external concentration polarization in both sides of the draw and feed solution. J. Membr. Sci..

[28] Koseoglu H., Guler E., Harman B.I., Gonulsuz E. (2018). Water Flux and Reverse Salt Flux. Membrane-Based Salinity Gradient Processes for Water Treatment and Power Generation.

[29] Goi Y., Liang Y., Lau W., Weihs G.F. (2023). Analysis of the effect of advanced FO spacer on the specific energy consumption of hybrid RO desalination system. J. Membr. Sci..

[30] Sekino M. (1993). Precise analytical model of hollow fiber reverse osmosis modules. J. Membr. Sci..

[31] Yip N.Y., Tiraferri A., Phillip W.A., Schiffman J.D., Hoover L.A., Kim Y.C., Elimelech M. (2011). Thin-Film Composite Pressure Retarded Osmosis Membranes for Sustainable Power Generation from Salinity Gradients. Environ. Sci. Technol..

[32] Jung D.H., Lee J., Kim D.Y., Lee Y.G., Park M., Lee S., Yang D.R., Kim J.H. (2011). Simulation of forward osmosis membrane process: Effect of membrane orientation and flow direction of feed and draw solutions. Desalination.

[33] Shibuya M., Yasukawa M., Goda S., Sakurai H., Takahashi T., Higa M., Matsuyama H. (2016). Experimental and theoretical study of a forward osmosis hollow fiber membrane module with a cross-wound configuration. J. Membr. Sci..

[34] Gaiser G. (2013). Kombinierte Wärme-und Stoffübertragung in Rotierenden Regeneratoren. VDI-Wärmeatlas: Mit 320 Tabellen.

[35] Munubarthi K.K., Gautam D.K., Reddy K.A., Subbiah S. (2020). Distributed parameter system modeling approach for the characterization of a high flux hollow fiber forward osmosis (HFFO) membrane. Desalination.

[36] Vitagliano V., Lyons P.A. (1956). Diffusion Coefficients for Aqueous Solutions of Sodium Chloride and Barium Chloride. J. Am. Chem. Soc..

[37] Sanahuja-Embuena V., Khensir G., Yusuf M., Andersen M.F., Nguyen X.T., Trzaskus K., Pinelo M., Helix-Nielsen C. (2019). Role of Operating Conditions in a Pilot Scale Investigation of Hollow Fiber Forward Osmosis Membrane Modules. Membranes.

